# Spherical submicron YAG:Ce particles with controllable particle outer diameters and crystallite sizes and their photoluminescence properties†

**DOI:** 10.1039/d1ra04800g

**Published:** 2021-09-10

**Authors:** Asep Bayu Dani Nandiyanto, Yusuke Kito, Tomoyuki Hirano, Risti Ragadhita, Phong Hoai Le, Takashi Ogi

**Affiliations:** Departemen Kimia, Universitas Pendidikan Indonesia Jl. Dr Setiabudhi No. 229 Bandung 40154 Indonesia; Chemical Engineering Program, Graduate School of Advanced Science and Engineering, Hiroshima University 1-4-1 Kagamiyama Higashi-Hiroshima City Hiroshima 739-8527 Japan ogit@hiroshima-u.ac.jp +81-82-424-3765 +81-82-424-3765

## Abstract

The purpose of this study was to demonstrate the preparation of spherical submicron YAG:Ce particles with controllable particle outer diameters and crystallite sizes and their photoluminescence (PL) properties, which were produced using a flame-assisted spray-pyrolysis method followed by the annealing process. The correlation of particle outer diameter, crystallite size, and PL performance of the prepared particles was also investigated. Experimental results showed that the increases in the particle outer diameters have an impact on the obtainment of higher PL performance. Large particle outer diameters permitted the crystallites to grow more, whereas this is in contrast to the condition for small particle outer diameter having limitations in crystallite growth. This study also found that too large outer diameter (>557 nm) was not effective since crystallites cannot grow anymore and it permits possible scattering problems. This study provides significant information for optimizing synthesis parameters for controlling particle outer diameters and crystallite sizes, which could be relevant to other functional properties, especially for lens, solar cell, and LED applications.

## Abstract 2

We demonstrate the synthesis of spherical submicron YAG:Ce particles with controllable particle outer diameters and crystallite sizes and their photoluminescence properties, produced by a flame-assisted spray-pyrolysis method with annealing process.

## Graphical abstract

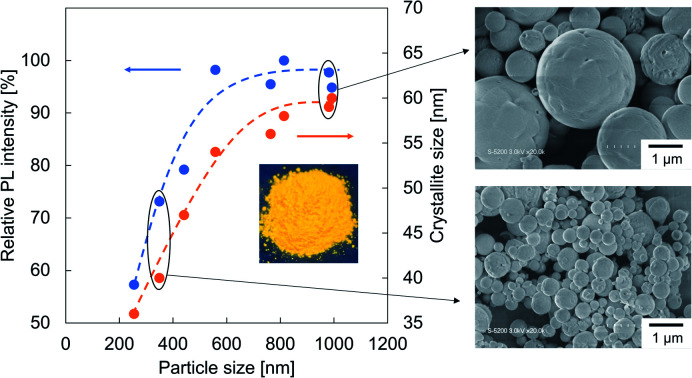

## 1. Introduction

Effective photoluminescence (PL) designs have attracted a lot of attention. To obtain excellent PL performance, several parameters must be considered:

(i) Surface roughness and particle morphology. The formation of particles with spherical morphology is very important in the synthesis of PL particles to obtain high brightness and high resolution. It is well known that spherical particles are more important because of their higher packing density, lower light scattering, and brighter luminescence performance.^1^

(ii) Particle outer diameter. The purity of the emission color becomes dependent on the particle outer diameter.^2,3^

(iii) Composition of crystallinity and crystallite size.^4,5^

(iv) The stoichiometric composition ratio of precursors in the synthesis process.^1,6^

(v) Doping component and heat treatment. Defects are created by adding a certain amount of impurity or doping. In addition, defect structures in the material can be made through heat treatment.^1,7^

Although the above parameters have been well-documented, researches on the particle outer diameter and particle shape, primarily with spherical morphology, on PL performance are still limited. Several studies have reported that the particle outer diameter has influences on improving PL performance. Wang *et al.*^4^ reported the effect of particle outer diameter on the PL spectra, showing a significant increase in PL intensity as the increases in the particle outer diameter. He *et al.*^8^ reported that the particle outer diameter (controlled with the addition of surfactants and pH conditions during the particle precipitation) showed a positive impact on the increases in PL intensity. Similar results were also reported by Hayashi^9^ using the particles synthesized using the hydrothermal method with various reaction temperatures. However, some problems are persisted in the obtainment of controlling particle morphology in the spherical shapes and having homogeneous sizes in the range submicron scale. Conventional materials for PL applications are usually in bulk forms with large sizes (from micrometer to millimeters). To further improve the PL materials' properties and apply them for ultra-small devices, it is necessary to construct PL particles with sizes in the submicron range with a spherical shape, which, however, are not easily synthesized. Although PL materials with nanometer sizes are possibly produced, the sizes of nano potentially limit the growth of crystal.

**Fig. 1:**
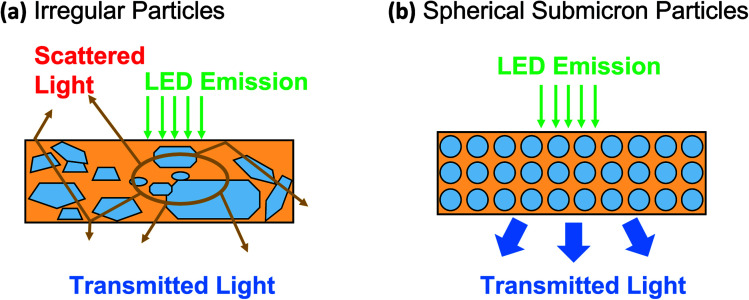
Performance comparison of commercially available YAG particles (a) and spherical YAG particles (b).

Fig. 1 shows the importance of spherical particles with homogeneous shapes in PL applications. In general, most commercial PL materials have irregular sizes and shapes (see Fig. 1(a)),^10,11^ bringing disadvantages, especially in the limitation when applied as light-emitting diode (LED) materials in the obtainment of a non-optimal light product.^12–14^ On the contrary, when the material was formed from homogenous submicron particles, the optimal LED material in the light absorption and transmission can be obtained (see Fig. 1(b)). However, to the best of our knowledge, studies on controlling particle outer diameter in the submicron range are still limited. The submicron-scaled particles can give better performance compared to nano and bulk materials.

Our previous studies^4,14–25^ reported the methods for preparing submicron particles with controllable particle outer diameters and crystallinities. Here, the purpose of this study was to demonstrate the synthesis route for the preparation of spherical submicron yttrium aluminum garnet (YAG):Ce particles with controllable particle outer diameters and crystallite sizes using a flame-assisted spray-pyrolysis method followed by the annealing process. The PL performance of the synthesized particles was also tested, and the influences of particle outer diameter and crystallite size on the PL performance of the prepared particles were investigated. YAG:Ce particles were selected because this material is prospective for being used in light utilization.^26^ YAG:Ce material has the characteristics of a relatively high absorption efficiency of blue excitation radiation (good optical properties).^26–28^ In addition to its optical properties, YAG:Ce material is also supported by extraordinary chemical stability and resistance to high temperatures.^29^ In terms of its unique properties, YAG:Ce material is intensively utilized in various applications, including cathode ray tubes, scintillators, electroluminescent displays, and field emission displays.^30–32^ The most important application of YAG:Ce material is often used to convert blue light-emitting diode (LED) radiation into a very wide band of yellow emission, which is employed to produce white light with gallium nitride-based blue LEDs.^23,26,33^

Many strategies for synthesizing YAG:Ce particles have been reported. Conventionally, YAG:Ce particles were prepared by mixing and calcining at 1600 °C precursor containing yttrium, alumina, and ceria. However, problems arose from the need of the high temperature around 1600 °C. Other methods are wet chemical route methods such as sol–gel,^28^ co-precipitation,^34^ solvothermal,^5^ and combustion methods.^35^ The synthesis route by solvothermal shows a good ability in producing excellent products,^36^ but the use of an autoclave that is done under a pressure condition of about 70–175 MPa remained some shortcomings. The combustion route is effective to produce particles in the rapid process but it has limitations in the inhomogeneity and agglomeration of the product.^37^ The sol–gel and co-precipitation methods are effective to conduct reactions at low temperatures and produce homogeneous particles.^28^ But, they have limitations in the need for multistep processes, and additional purification processes to get a high purity of products are mandatory. To against the above limitations, spray pyrolysis is one of the excellent methods.^17,38^ The produced particles are uniform in particle outer diameter, agglomeration-free product, and spherical-shaped particles.^21^ The spray pyrolysis is a rapid process, and the reaction occurs in the micron scales, which takes place in small droplets, allowing the produced particles in submicron to micrometer range.^16,18^

**Fig. 2:**
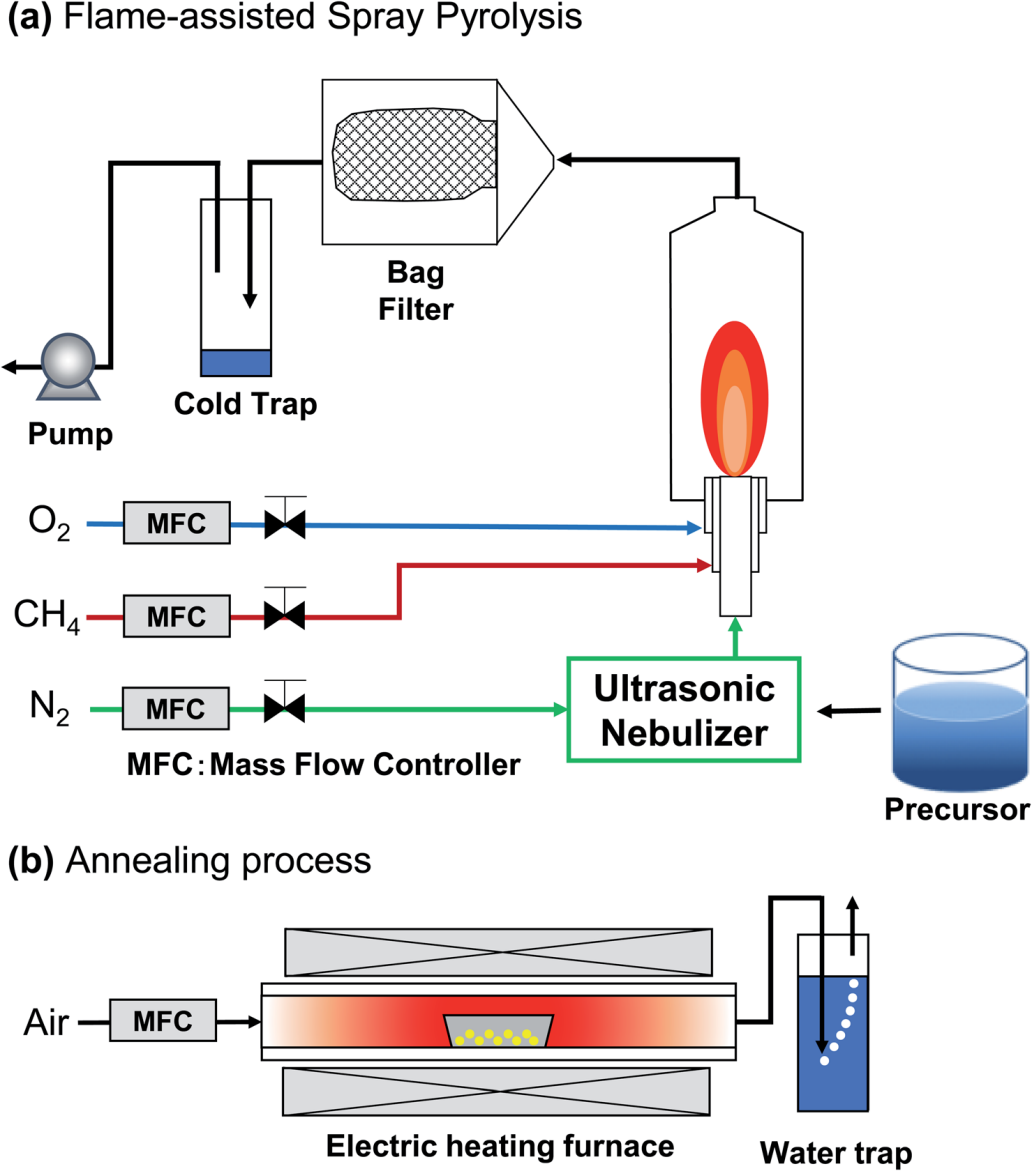
Illustration of YAG:Ce synthesis process using flame-assisted spray pyrolysis (a) combined with annealing process (b).

As a continuation of our previous study,^4^ synthesizing PL particles using the spray-pyrolysis, here the focus of this study was to demonstrate the use of flame-assisted spray pyrolysis followed with the annealing process method for producing spherical submicron YAG:Ce particles and to evaluate the correlation of particle outer diameter, crystallite size, and PL performance of the prepared particles. Different from Wang *et al.* method,^4^ the experiments were done in two steps (Fig. 2). In the first step (Fig. 2(a)), the flame-assisted spray-pyrolysis method was used to synthesize yttrium aluminum hexagonal (YAH) particles. In the second step (Fig. 2(b)), an additional annealing process was used to convert YAH particles into YAG particles. The produced particles were spherical, which are effective for being packed and assembled for further uses such as LEDs. Different from previous studies, the novelties of this study were the successful preparation of spherical submicron particles and the investigation of PL particles' performance in the range of submicrometer (between 200 and 1200 nm).

## 2. Material and method

### 2.1. Materials

The synthesis of YAG:Ce particles used three main precursors: yttrium(iii) nitrate hexahydrate (Y(NO_3_)_3_·6H_2_O), aluminum nitrate nonahydrate (Al(NO_3_)_3_·9H_2_O), and cerium(iii) nitrate hexahydrate (Ce(NO_3_)_3_·6H_2_O) as sources of Y, Al, and Ce, respectively. All chemicals were purchased from Sigma-Aldrich Co. Ltd., USA and used without further purification.

### 2.2. Method

The synthesis of YAG:Ce particles was carried out in two steps (see Fig. 2): (1) flame-assisted spray pyrolysis and (2) annealing process. Before the flame-assisted spray pyrolysis stage was carried out, the precursor mixture solution was prepared by mixing yttrium nitrate hexahydrate, aluminum nitrate nonahydrate, and cerium nitrate hexahydrate in ultrapure water at room temperature for 30 minutes. The concentration of the precursor mixture solution was set at 0.01, 0.05, 0.10, 0.30, 0.50, 0.80, 1.00, and 1.20 mol L^−1^.

In the first stage, the precursor was put into the flame-assisted spray-pyrolysis apparatus (see Fig. 2(a)). The apparatus consisted of an ultrasonic nebulizer, a diffusion flame burner, a glass reactor, and a bag filter. The apparatus was used for converting precursor solution into droplets, as well as evaporating the solvent and reacting component to YAH. Methane and oxygen were used as the fuel and the oxidizer, respectively. The methane and oxygen gas flow rates were set at 2 and 5 L min^−1^, respectively. In short, the precursor solution mixture was atomized using an ultrasonic nebulizer at 1.7 MHz (NE-U17, Omron Healthcare Co., Ltd., Tokyo, Japan), and the generated droplets were subsequently fed into the central tube of the burner with a carrier gas. The flow of N_2_ carrier gas was fixed at 3 L min^−1^ to ensure the mass and heat transfer processes can be done completely, preventing issues in the formation of non-spherical droplets.^39^ The as-prepared particles were collected in a bag filter. During the experiment, the temperature of the particle collector was maintained at 120 °C to avoid water condensation.

In the second step (see Fig. 2(b)), the obtained particles from the flame-assisted spray pyrolysis process were converted into YAG particles through the annealing process for 2 hours at 1200 °C under a heating rate of 10°C min^−1^ and air gas flow rate at 1 L min^−1^.

### 2.3. Characterization of spherical submicron YAG particles

The crystalline structures of the prepared samples were characterized using an X-ray powder diffraction (XRD; D2 PHASER, 40 kV and 30 mA, Bruker Corp., U.S.A.) with Cu Kα radiation. The crystal sizes (*d*_c_) were calculated from the XRD peak at 34° using the Scherrer equation:^40^
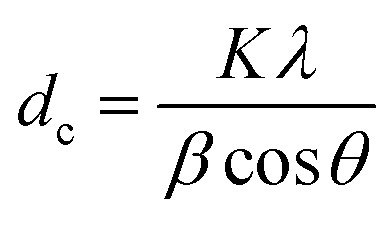
where *K* (= 0.89) is the shape factor, *λ* (= 0.154 nm) is the X-ray wavelength, *β* is the line broadening at half the maximum intensity in radians, and *θ* is the Bragg angle. Detailed calculation is explained elsewhere.^40^

The morphologies of the prepared powders were observed using a scanning electron microscope (SEM; S-5200, Hitachi, Tokyo, Japan; operated at 3 kV). The particle outer diameters (*d*), as the Feret diameter, were directly measured by counting approximately 500 particles in SEM images. Detailed information about Feret analysis was explained in literature.^41^ The quantum efficiency was analyzed using a PL quantum efficiency measurement system (C9920-02, Hamamatsu Photonics, Shizuoka, Japan).

The interior morphologies of the prepared phosphor particles were observed using a transmission electron microscope (TEM; JEM-2010, JEOL Ltd., Tokyo, Japan; operated at 200 kV). The PL spectra of the obtained YAG:Ce particles were measured at room temperature using a spectrofluorophotometer (RF–5300PC, Shimadzu, Kyoto, Japan) equipped with a Xe lamp as the light source. All PL analyzes were conducted at room temperature under excitation at 468 nm.

## 3. Results and discussion

**Fig. 3:**
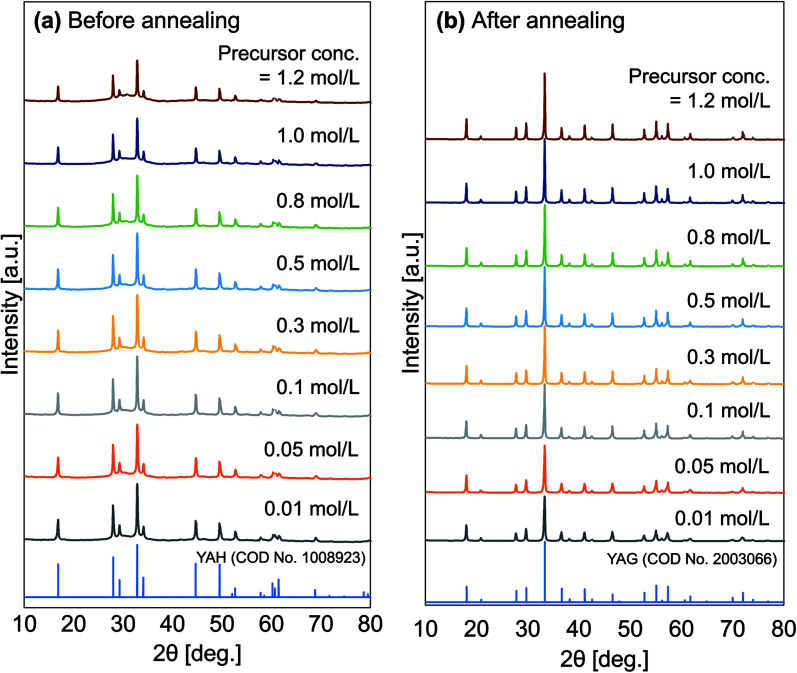
XRD patterns of the obtained particles before (a) and after (b) the annealing process for all various initial concentration.

Fig. 3 shows the XRD patterns of particles produced at different initial precursor concentrations taken before and after the annealing process. Changes in the XRD patterns were detected. Fig. 3(a) shows that spectra for samples with certain initial precursor concentrations, in which the results were in accordance with pure YAH based on the crystallography open database (COD) no. 1008923. YAH phase is a metastable form in the Y_2_O_3_–Al_2_O_3_ system. After the annealing process (Fig. 3(b)), all samples undergo conversion from YAH to YAG. The XRD diffraction patterns of annealed samples show compatibility with pure YAG based on COD no. 2003066. All diffraction patterns of the samples, both before (YAH particles) and after experiencing the annealing process (YAG:Ce particles) showed no impurity peaks. No transitions to other phases were detected, indicating that the product is pure in the YAG crystalline phase. In the annealed samples, the intensities of the XRD patterns increased with increasing precursor concentrations. The high initial precursor concentration led to the formation of better crystallinities because the high initial precursor concentration associates with a large outer diameter formation,^42^ giving a large space for the crystal to do more growth and resulting in larger crystallite sizes.^27,43^ Based on the Scherer equation, the annealed samples produced with the initial precursor concentrations of 0.01, 0.05, 0.10, 0.30, 0.50, 0.80, 1.00, and 1.20 mol L^−1^ had crystal sizes of 36, 40, 47, 54, 56, 58, 59, and 60 nm, respectively.

The reason for the crystal transformation is described in the following. During the spray pyrolysis process, phenomena occur among components of yttrium nitrate hexahydrate, aluminum nitrate nonahydrate, and cerium nitrate hexahydrate inside the droplet. In the flame-assisted spray pyrolysis process, the decomposition process happens in the carbon group, resulting in the removal of nitrate from the precursor. Thus, most of the carbon, nitrogen, and hydrogen elements are lost from the precursor. Such removal can lead to the formation of metal oxides. The result of the synthesis of the spray pyrolysis process is YAlO_3_ (or YAH) particles, which are metastable forms in the Y_2_O_3_–Al_2_O_3_ system.

In the annealing process, the YAH phase converted to YAG phase.^44^ Using the annealing process with temperatures of higher than 1100 °C, a crystalline YAG was formed.^45^

**Fig. 4:**
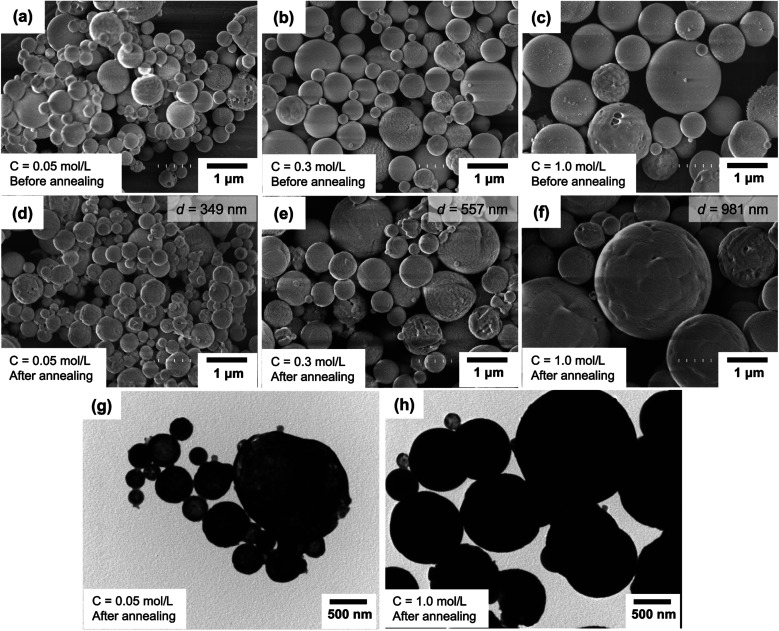
SEM images of the particles prepared using the initial precursor concentration of 0.05 (a and d); 0.3 (b and e); and 1.00 mol L^−1^ (c and f) before (a–c) and after (d–f) the annealing process. (g and h) are the TEM images of particles prepared using an initial precursor of 0.05 and 1.00 mol L^−1^, respectively.

Fig. 4(a)–(c) and (d)–(f) present SEM images of particles before and after the annealing process, respectively. Agglomeration-free spherical particles were found for all variations of the initial precursor concentrations. Changes in the outer diameters for samples before and after the annealing process were found, and the main parameters are due to the change in the initial precursor concentration. The mean outer diameters of the annealed particles using the initial precursor concentrations of 0.05, 0.30, and 1.00 mol L^−1^ were 349, 557, and 981 nm, respectively. During the droplet evaporation process in flames, a large loss of solvent from the droplet occurs, remaining main components only in the final product. Indeed, when a low concentration is in the droplet, the remaining components after the evaporation should be in a small number, resulting in a small particle outer diameter.^42^ This leads to the fact that high concentration relates to the formation of a larger outer diameter, informing the potential control of particle outer diameter by changing the initial precursor concentration.

Fig. 4(g) and (h) present the TEM images of annealed particles prepared using initial precursor concentrations of 0.05 and 1.00 mol L^−1^, corresponding to the obtainment of outer diameters/crystallite sizes of about 349/40 and 981/59 nm, respectively. Based on the TEM observations, Fig. 4(g) shows that particles with relatively smaller crystallite sizes (40 nm) have less dense structures. The particles have incomplete spherical morphologies. Fig. 4(h) presents particles with relatively larger crystallite sizes (59 nm), a dense structure, and excellent spherical morphology.

**Fig. 5:**
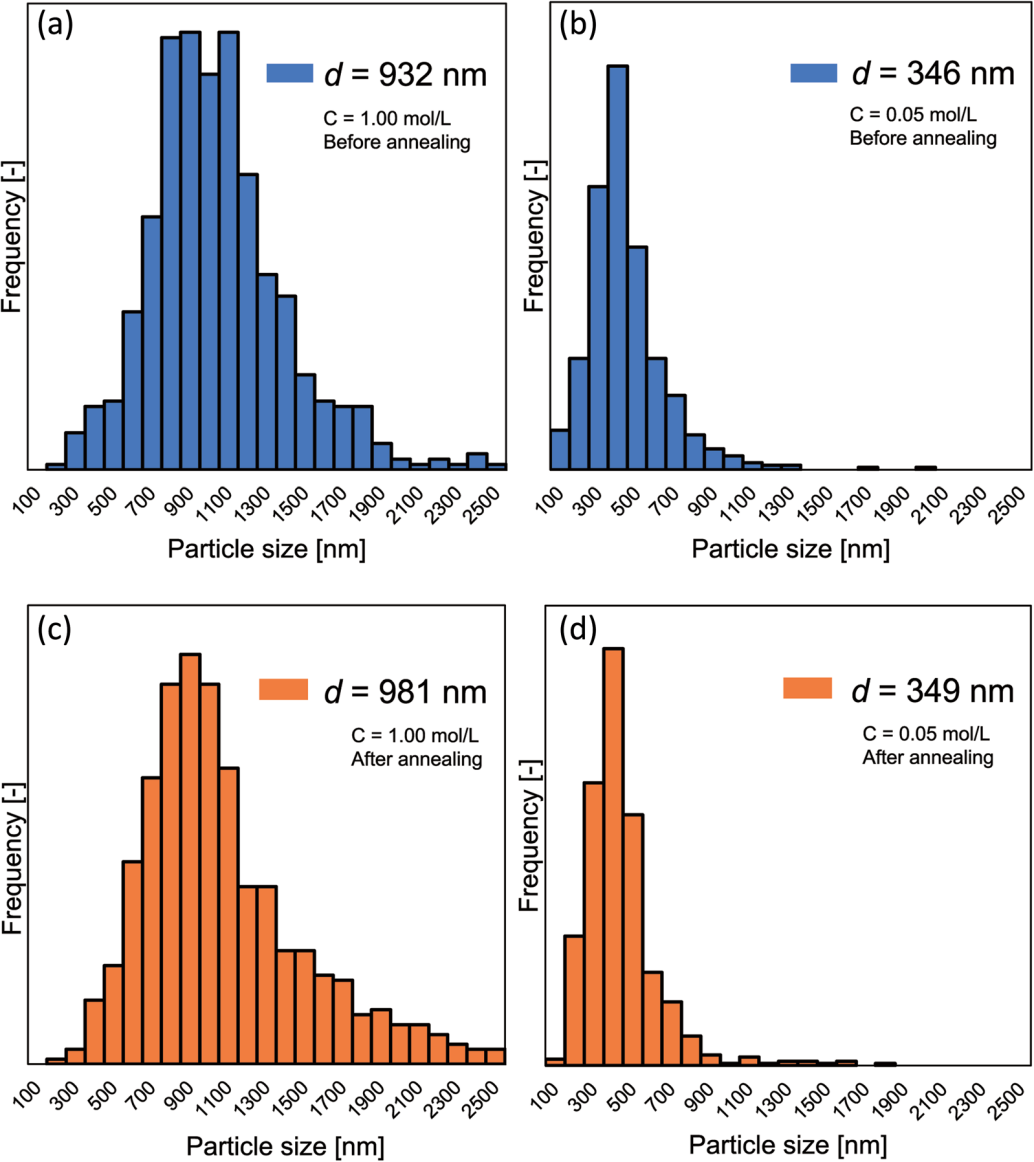
Particle size distribution of the particles prepared using the initial precursor concentration of 1.00 (a and c) and 0.05 (b and d). (a and b) and (c and d) are particle size distributions for before and after the annealing process, respectively.

Fig. 5(a)–(d) show the detailed changes in the particle outer diameter before and after the annealing process, respectively. No change in particle outer diameter distribution was observed before and after the annealing process. After the annealing process, two samples with the initial concentrations of 0.05 and 1.00 mol L^−1^ had the mean particle outer diameter of 349 and 981 nm, respectively.

**Fig. 6:**
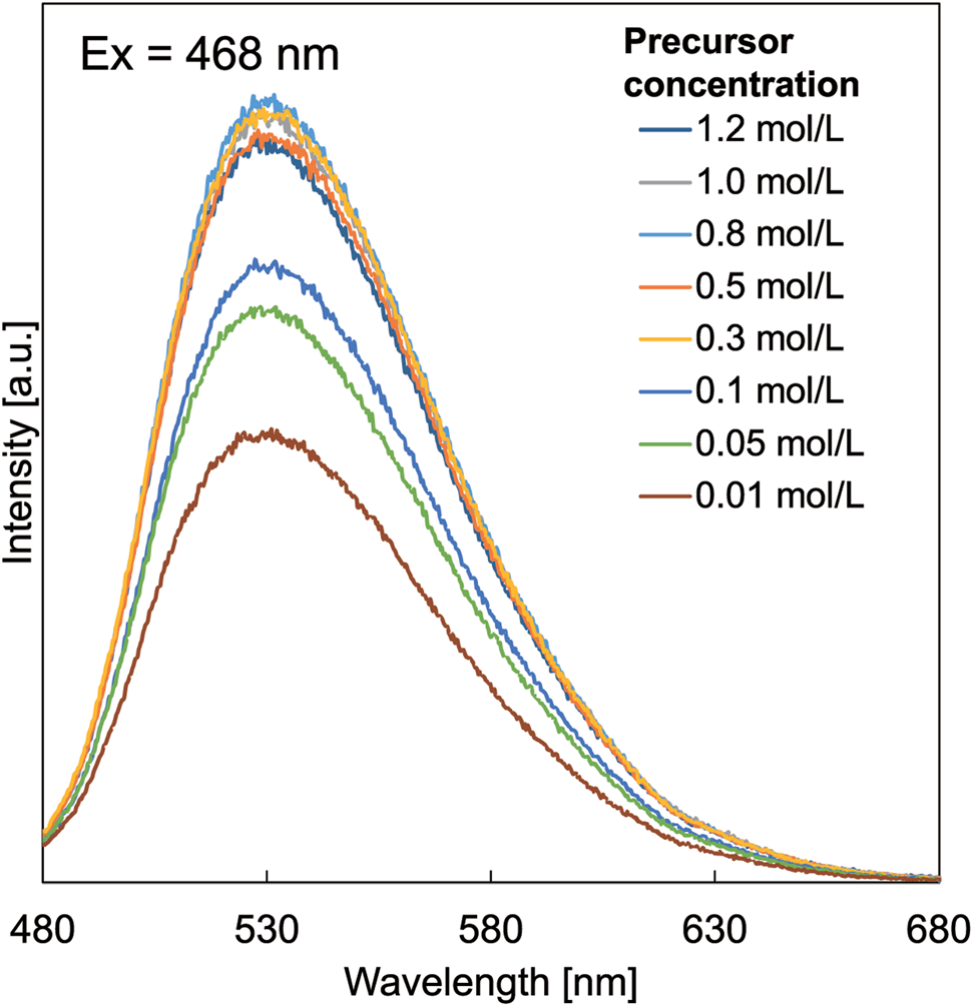
PL emission spectra of YAG:Ce particle.

Fig. 6 shows the effect of particle outer diameter on PL emission spectra of prepared particles with various initial precursor concentrations. Samples with certain initial precursor concentrations were tested by excitation at a wavelength of 468 nm, producing a broad-emission spectrum in the wavelength of 480–680 nm. For all samples, the maximum emission peak (measured the excitation peak at an emission of 530 nm without a visible shift assigned to the 5d (^2^A_1g_) → 4f (^2^F_5/2_ and ^2^F_7/2_) transitions of Ce^3+^, since Ce^3+^ with 4f1 electron configuration) has two ground state of ^2^F_5/2_ and ^2^F_7/2_, informing a spin–orbit interaction.^46^ Based on the PL spectra, increases in the initial precursor concentrations caused increases in the intensities of the emission band and decreases in surface defects.^47^ Initial precursor concentration influences the PL intensity, which is due to the fact that the initial precursor gives impacts on the formation of crystallite sizes as confirmed in the XRD analysis results (Fig. 3). The existence of the effect of crystallite size on the PL intensity in this study is in good agreement with the literature.^25^ In addition, PL emission spectra of YAH were measured (see Fig. SI-1 in the ESI†). However, no PL peak was observed in the YAH particles, which is reasonable since YAH is not a phosphor material.

**Fig. 7:**
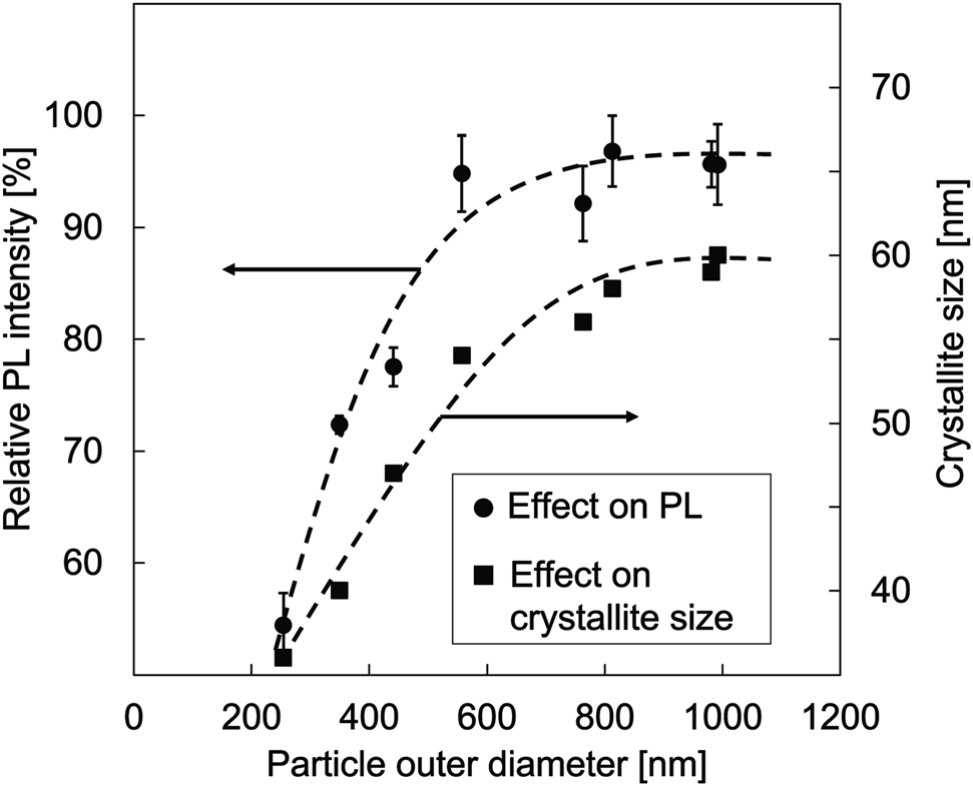
The relationship of particle outer diameter, crystallite size, and PL intensity of YAG:Ce particles.

Fig. 7 shows the correlation of particle outer diameter, crystallite size, and PL intensity of the prepared YAG:Ce particles. To ensure the analysis for the PL intensities, an error bar was added for each measurement.

The relationship between the particle size and the PL intensity as shown in Fig. 7 confirmed significant increments of PL intensities as the increases in the particle outer diameters from 254 to 557 nm. However, when the particle outer diameter was greater than 557 nm, no significant increases in the PL intensities were found. The PL intensities tended to be saturated.

In the case of particle outer diameter of smaller than 557 nm, the increases in the PL intensities occur because of the contribution of crystallite size in the particle. The high initial precursor concentration leads to the formation of better crystallinities. The high initial precursor concentration associates with a large outer diameter formation, giving a large space for the crystal to do more growth and resulting in the creation of larger crystallites.^27,43^ This is in contrast to the condition for small particles, having limitations in crystallite growth. However, too high concentration has no impact on the crystal growth. Indeed, to make further crystal growth, additional treatment must be added. Regarding the case in the particle outer diameter of larger than 557 nm, there are constant PL intensities because the scattering of incident light inside particles tends to increase.^4^

**Fig. 8:**
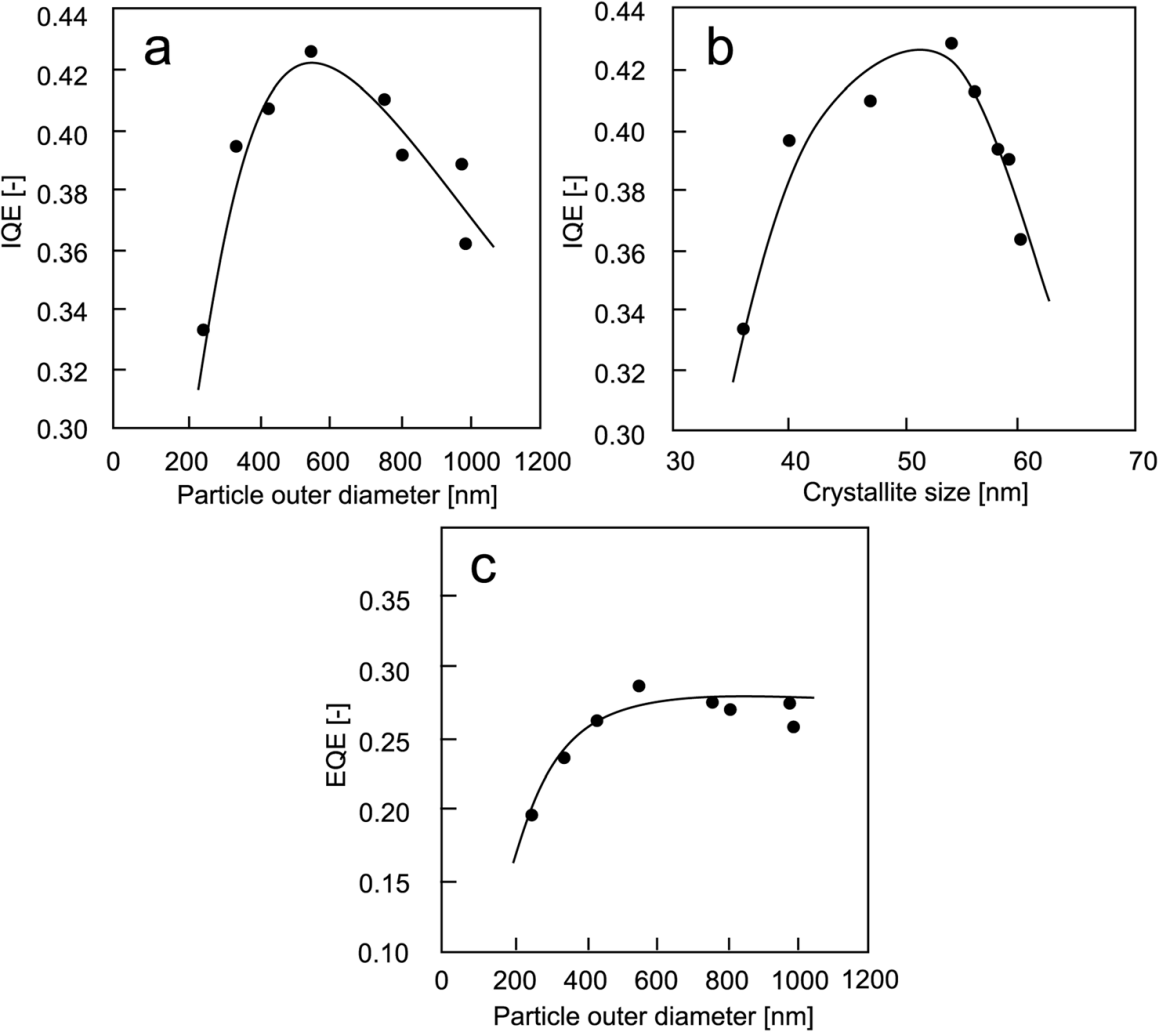
The QE results: (a) IQE *versus* particle outer diameter; (b) IQE *versus* crystallite size; (c) EQE *versus* particle outer diameter.

The results from QE experiments are presented in Fig. 8. The internal quantum efficiency (IQE) results were classified as the effect of particle outer diameter (Fig. 8(a)) and crystallite size (Fig. 8(b)). Fig. 8(c) is the external quantum efficiency (EQE) results as a function of particle outer diameter.

Table 1 summarizes the correlation of the initial precursor concentration, the resulting particle outer diameter (*d*) and the crystallite size (*d*_c_) based on the SEM and XRD results, respectively. The table is also completed with the IQE and the absorption rate. The results showed that the increases in the initial precursor concentrations gave an impact on the preparation of larger particle outer diameters and larger crystallites, and the changes affected the IQE and the absorption rate.

**Table 1:** Effect of initial concentration of precursor on the final particle outer diameter and crystallite size as well as IQE and absorption rate

Precursor initial concentration (mol L^−1^)	Particle outer diameter *d* (nm)	Crystallite size *d*_c_ (nm)	Internal quantum efficiency (−)	Absorption rate (−)
0.01	245	36	0.334	0.59
0.05	349	40	0.396	0.59
0.10	441	47	0.409	0.64
0.30	557	54	0.428	0.67
0.50	763	56	0.412	0.67
0.80	813	58	0.393	0.69
1.00	981	59	0.390	0.71
1.20	992	60	0.363	0.71

When particle outer diameter is smaller than 600 nm (Fig. 8(a)), the internal quantum efficiency (IQE) increases with increasing the particle outer diameter. However, measuring IQE for the particle outer diameter of larger than 600 nm, opposite results were obtained. The larger particle outer diameter brings a negative influence to the obtainment of IQE value.

Regarding the crystallite size of less than 55 nm (Fig. 8(b)), the IQE results increase with increasing crystallite sizes. The opposite tendency was obtained for particles with too large crystallite sizes.

The correlation between EQE and particle outer diameter is presented in Fig. 8(c). The results showed that the tendency of the curve is in good agreement with the PL results in Fig. 7. The increases in the EQE value happen only when using particle outer diameters of less than 600 nm. Then, the EQE value is almost constant when using larger particles.

In short, the absorption of photon shifts to lower energy as the outer diameter of the particle increases (see Table 1), which is due to a decrease in the quantum confinement.^48^ The larger particles had higher absorption rates. The IQE increases with increasing particle outer diameter and crystallite size. However, a decrease in IQE after reaching a certain particle outer diameter (>550 nm) and crystal size (>55 nm) is due to an increase in the adsorption rate of particle (Table 1).^4^

IQE and EQE are important characteristics. The IQE is the ratio between the number of photons emitted internally and the photons absorbed in the sample layer of the luminescence material. Usually, IQE is always greater than EQE. A low IQE indicates that the phosphor cannot utilize photons well. High IQE can be transformed into an impressive EQE.^49^ The IQE follows eqn (1).1
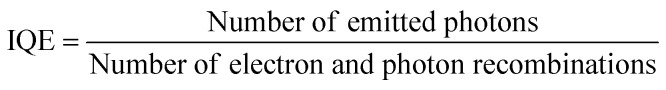


Meanwhile, EQE is the ratio between the number of photons emitted externally and the total incident photons in the luminescence material sample layer. The EQE of material includes optical loss effects such as transmission and reflection. However, it is often useful to look at the quantum efficiency of the remaining light after the reflected and transmitted light has disappeared. The EQE follows eqn (2).^49^2
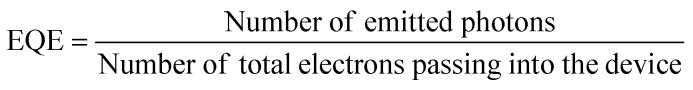


The results in Fig. 8 confirm the approach to improve the PL quantum yield characteristics, which is by optimizing the particle outer diameter and crystal size for reducing the rate of nonradiative recombination, which is in good agreement with literature by minimizing bulk as well as controlling surface and interfacial defects.^50^

**Fig. 9:**
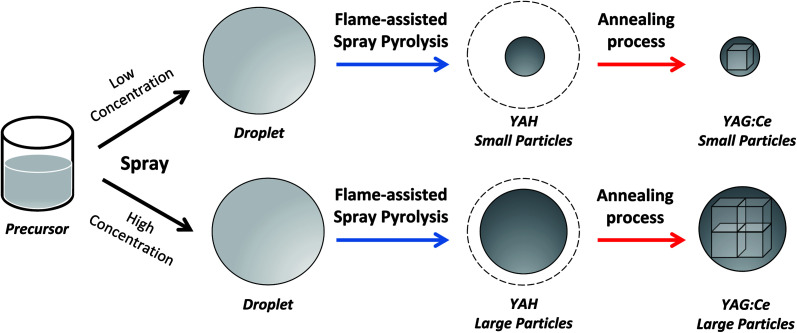
Proposal illustration of the formation of particles with controllable particle outer diameters and crystallite sizes.

Fig. 9 depicts the proposal illustration of the formation of crystallites in this study. In short, the initial process is done by spraying a precursor to form droplets. Assuming no change in the droplet morphology during the pyrolysis,^39^ the outer diameter of the as-synthesized particles is a function of the initial concentration of precursor. A low concentration of precursor allowed the formation of small particles, whereas a high concentration led to the production of large particles.^42^ Finally, an additional annealing process to the spray-pyrolyzed particles promotes the conversion of YAH into YAG crystal inside the particle. Interestingly, the large particle outer diameter permits the crystallites to grow more. This is in contrast to the condition for the small particle outer diameter having limitations in the crystallite growth. Indeed, this makes large particles have large crystallites.

In addition, this study evaluated the one-sided effect by adjusting initial precursor concentration for controlling outer diameter. Then, the outer diameter gives an impact on the crystallite sizes. The larger outer diameter promotes the creation of larger crystallite sizes.

The most conductive in the crystallization is in the second step of the preparation procedure, *i.e.* the annealing process. The annealing process involves three aspects: (i) converting YAH into YAG, (ii) allowing nucleation process, and (iii) providing crystal growth.

The annealing process was fixed in the condition at 1200 °C for 2 hours, which is a good condition for the crystallites to grow optimally based on our previous study.^25^ Although the increases in the temperature have correlations to the formation of larger crystallites, there are issues in the sintering and change in the emission peak. Too high temperature tends to shift the emission peak to the blue region. This blue shift originates from the reduction of the cell parameter inside the lattice, implying a smaller atomic spacing and a stronger ligands field of YAG.^25^ In addition, further investigations of the effect of annealing time and temperature on promoting control of crystallite size and PL properties are important and will be done in our future work.

In addition, another analysis that should be considered is the lifetime experiments. The fluorescence lifetime is a function of particle characteristics (*e.g.* particle size, crystallite size, annealing temperature, *etc.*), and detailed experiments will be conducted in our future work.

## 4. Conclusion

The spherical submicron YAG:Ce particles have been successfully prepared using the flame-assisted spray pyrolysis followed by the annealing process. The effects of particle outer diameter and crystallite size on the PL performance of the submicron spherical YAG:Ce particles have been also observed. Variations in the initial precursor concentrations are made to control the YAG:Ce particle outer diameter and crystallite size. The PL performance of the synthesized submicron-sized spherical YAG:Ce particles showed increases in intensity values when the particle outer diameter and crystallite size increased as well. Particle outer diameter and crystallite size play important roles in improving the PL performance of YAG:Ce particles. The large particle outer diameter permitted the crystallites to grow more. This is in contrast to the condition for the small particle outer diameter having limitations in crystallite growth. However, too large particle outer diameter (>557 nm) was not effective since crystallites cannot grow anymore as well as the issues in the light scattering possibly exist.

## Conflicts of interest

We certify that all authors agree for publishing this work, and we have no conflict of interest to declare. We warrant that the article is the authors' original work.

## Supplementary Material

RA-011-D1RA04800G-s001
