# Supplementary material for: Spherical submicron YAG:Ce particles with controllable particle outer diameters and crystallite sizes and their photoluminescence properties
Source: RSC Adv. 2021 Sep 10;11(48):30305–14. doi: 10.1039/d1ra04800g (PMC9041146; doi:10.1039/d1ra04800g)
Supplement: RA-011-D1RA04800G-s001 [file RA-011-D1RA04800G-s001.pdf]

Electronic Supplementary Material (ESI) for RSC Advances.
This journal is © The Royal Society of Chemistry 2021

Supporting Information

Spherical Submicron YAG:Ce Particles with Controllable Particle Outer Diameters and Crystallite Sizes and Their Photoluminescence Properties

Asep Bayu Dani Nandiyanto[1], *Yusuke Kitou*[2], *Tomoyuki Hirano*[2], *Risti Ragadhita*[1], *Phong Hoai Le*[2], *and Takashi Ogi*[2*]

[1]Departemen Kimia, Universitas Pendidikan Indonesia, Jl. Dr. Setiabudhi No. 229, Bandung 40154, Indonesia.

[2]Chemical Engineering Program, Graduate School of Advanced Science and Engineering, Hiroshima University, 1-4-1 Kagamiyama, Higashi-Hiroshima City, Hiroshima 739-8527, Japan.

*Corresponding Author: Takashi Ogi

E-mail: ogit@hiroshima-u.ac.jp

Tel/ Fax: +81-82-424-3765

Chemical Engineering Program, Graduate School of Advanced Science and Engineering, Hiroshima University, 1-4-1 Kagamiyama, Higashi-Hiroshima City, Hiroshima 739-8527, Japan.

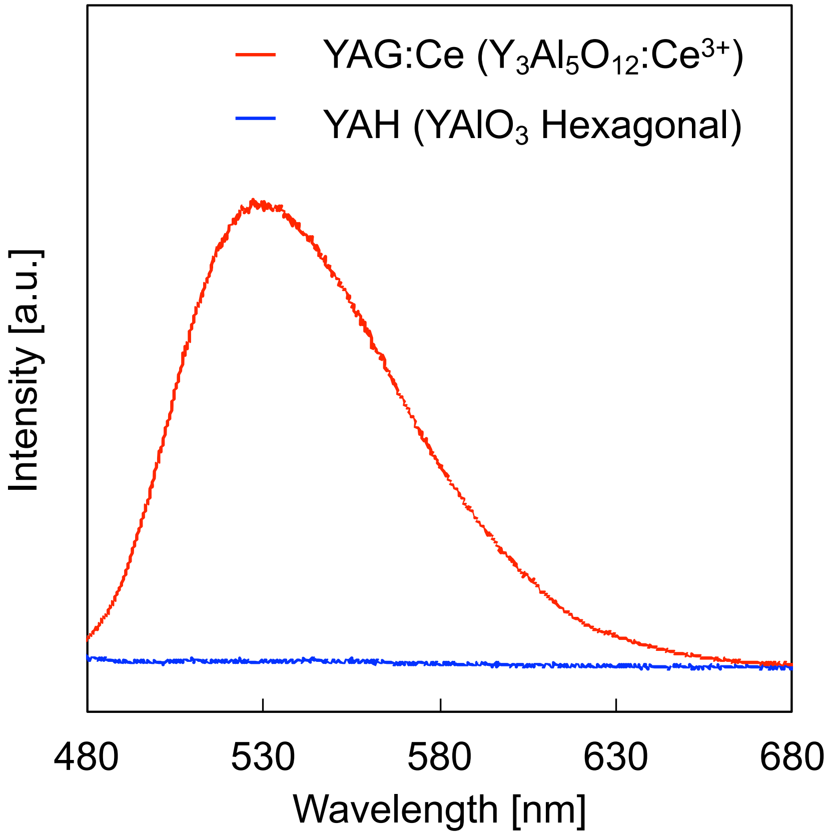

Figure SI-1. Comparison of PL intensities of YAG:Ce and YAH. YAG sample was done using 1.2 mol/L of precursor concentration.
